# In Vitro Study of the Interaction of Gentamicin with Ceftriaxone and Azithromycin against *Neisseria gonorrhoeae* Using Agar Dilution Method

**DOI:** 10.3390/antibiotics11081083

**Published:** 2022-08-10

**Authors:** Wenqi Xu, Qian Zhou, Jingwei Liu, Yan Zhang, Xiaoyu Zhu, Bangyong Zhu, Yueping Yin

**Affiliations:** 1 Institute of Dermatology, Chinese Academy of Medical Sciences and Peking Union Medical College, Nanjing 210042, China; 2 STD Reference Laboratory, National Center for Sexually Transmitted Diseases Control, Chinese Center for Disease Control and Prevention, Nanjing 210042, China; 3 Institute of Dermatology, Guangxi Autonomous Region, Nanning 530003, China

**Keywords:** *Neisseria gonorrhoeae*, synergy, gentamicin

## Abstract

The susceptibility to gentamicin of *N. gonorrhoeae* isolates collected in three Chinese provinces and the correlation among the MICs of gentamicin, azithromycin, and ceftriaxone were investigated in this study. The effects of combinations from those three antibiotics were also in the scope of this study to determine the efficacy of gentamicin as a combination therapeutic drug. The agar dilution method was used to measure the minimum inhibitory concentrations (MICs) of ceftriaxone, azithromycin and gentamicin on *N. gonorrhoeae* isolates. The synergy between these three antimicrobials were determined using the agar dilution checkerboard method. Subgroup studies were conducted to explore differences between azithromycin- and ceftriaxone-sensitive and resistant isolates. A total of 139 (36.60%) and 233 (61.30%) isolates demonstrated full susceptibility and intermediate susceptibility to gentamicin, respectively. The correlation analysis showed that the MICs of ceftriaxone and azithromycin weakly correlated with the value of gentamicin. The overall results of the three antibiotic combinations revealed indifferent effects. Combination therapy established a significant reduction on the MIC value. Most of the *N. gonorrhoeae* isolates tested in this study demonstrated a certain degree of susceptibility to gentamicin. Overall, antimicrobial combinations of gentamicin with ceftriaxone or azithromycin demonstrate indifferent effects.

## 1. Introduction

The pathogen *Neisseria gonorrhoeae* (*N. gonorrhoeae*) is responsible for gonorrhea, which induces urogenital, pharyngeal, or rectal mucosa infections in sexual transmission [1]. In 2016, a study released by the World Health Organization (WHO) estimated that there were 86.9 million gonorrhea cases worldwide among adults aged 15 to 49 years old [2]. Antibiotics are now the mainstay of therapy for *N. gonorrhoeae* infection [3]. Treatment guidelines developed in the United States, Europe, and by the WHO recommend dual therapy with azithromycin and ceftriaxone for the treatment of gonorrhea [4,5,6]. Notably, since the antibiotics susceptibility of *N. gonorrhoeae* is declining over time, reports of instances of multidrug-resistant (MDR) *N. gonorrhoeae* have been frequent in many countries [7,8,9]. Thus, detecting plausible alternative antibiotics and searching for novel treatment options are critical to hindering the progress of *N. gonorrhoeae* antibiotic resistance [10].

Gentamicin is a widely used aminoglycoside that inhibits bacterial protein synthesis in gram-negative bacteria by directly binding to the decoding A site of the bacterial 30S ribosomal subunit [11]. Gentamicin is currently used as an alternative therapy to ceftriaxone in the US, Europe, and according to the WHO guidelines [4,6,12]. In Malawi, the combination of gentamicin with doxycycline as first-line therapy has been applied to treat genitourinary infections caused by *N. gonorrhoeae* for nearly 30 years [13]. In China, gentamicin’s in vitro activity against *N. gonorrhoeae* was investigated by Liu et.al in 2017, which showed that 85.9% of isolates were fully sensitive to gentamicin [14]. However, in a meta-analysis, the combined cure rate of single-dose gentamicin treatment was only 91.5% [15]. In another clinical study, the combination of gentamicin with azithromycin elicited a 100.0% cure rate in patients with uncomplicated gonorrhea [16]. Therefore, gentamicin is suggested to be more appropriate as an antimicrobial agent in a dual treatment regimen rather than as first-line empirical monotherapy [17]. Gentamicin can help decrease the progression of drug resistance when used in combination with ceftriaxone or azithromycin [18].

To promote gentamicin as an alternative antibiotic in China for *N. gonorrhoeae* infection, monitoring the susceptibility of *N. gonorrhoeae* isolates in China to gentamicin and antimicrobial combination testing for gentamicin with other antibiotics can be of great interest. In this in vitro study, the susceptibility to gentamicin of *N. gonorrhoeae* collected in three Chinese provinces from 2019 to 2020 was evaluated and an analysis of the correlation among the MICs of gentamicin, azithromycin and ceftriaxone was established. Ultimately, the effects of the three antimicrobial drug combinations were also investigated to determine the effectiveness of gentamicin in a combination drug therapy.

## 2. Methods

### 2.1. N. gonorrhoeae Isolates

From December 2019 to December 2020, 380 clinical strains of *N. gonorrhoeae* isolates were collected in three Chinese provinces: Tianjin (*n* = 110), Xinjiang (*n* = 167), and Zhejiang (*n* = 103)). All of the strains were previously determined to be *N. gonorrhoeae* through standardized methodologies [19]. The Medical Ethics Committee at the Institute of Dermatology, the Chinese Academy of Medical Sciences & Peking Union Medical College and the National Center for Sexually Transmitted Disease Control all gave their approval to this project (2014-LS-026). This Declaration of Helsinki guidelines were followed in this study. Study participants signed an informed consent form before inclusion in the study. Before the antimicrobial agent susceptibility tests, all of the strains were preserved in skim milk at −80 °C. The WHO reference strains G, P, O, J, and K were used as quality controls for MIC determinations.

### 2.2. Antimicrobial Susceptibility Testing

The antimicrobial susceptibility of all isolates to ceftriaxone, azithromycin, and gentamicin was evaluated using the WHO’s standard agar dilution method [19]. The drugs were obtained from Sigma Aldrich. Two operators independently read the results on the antibiotic plate at the same time, and in the case of a discrepancy, a third experimental supervisor made the result determination. We classified azithromycin’s MIC into susceptibility (MIC ≤ 0.5 mg/L) or resistance (MIC ≥ 1.0 mg/L), and that of ceftriaxone into susceptibility (MIC ≤ 0.125 mg/L) or resistance (MIC ≥ 0.25 mg/L). The criteria for azithromycin and ceftriaxone susceptibility and resistance were interpreted based on the Clinical and Laboratory standards Institute (CLSI) M100-S29 breakpoints [20]. According to previous studies and interpretation criteria, the breakpoint for gentamicin MIC was set to be ≤4 mg/L as fully susceptible, 8 to 16 mg/L as intermediately susceptible, and ≥32 mg/L as resistant [14,21].

### 2.3. Synergy Testing

To test the synergy between these three antimicrobials, the agar dilution checkboard method was used [22]. Gentamicin (0.5–32 mg/L in 7 two-fold dilutions) and azithromycin (0.015–8 mg/L in 10 two-fold dilutions), gentamicin (0.5–32 mg/L) and ceftriaxone (0.004–0.5 mg/L), azithromycin (0.015–8 mg/L) and ceftriaxone (0.004–0.5 mg/L) were added to the antimicrobial medium separately. The rest of the procedure was identical to the antimicrobial susceptibility testing.

### 2.4. Statistical Analysis

Descriptive statistics were used to characterize the MIC distributions. Furthermore, following logarithmic conversion of the MIC values, the correlation coefficient *R* was used to identify connections between the MIC of various antibiotics. Poor, medium and strong correlations correspond to *R* values in the range of 0.3–0.5, 0.5–0.8 and 0.8–1, respectively.

The fractional inhibitory concentration index (FICI) was used to assess the interactions. The FICI was calculated as (C_A_/MIC_A_) plus (C_B_/MIC_B_), where MIC_A_ and MIC_B_ are the individual MICs of antibiotics A and B, and C_A_ and C_B_ are their combined MICs, corresponding to the lowest FICI (or highest FICI, in the case of antagonism). When the FICI was less than 0.5; the interaction was categorized as synergistic; between 0.5 and 4.0, it was classified as indifferent; and when the FICI was greater than 4.0, the interaction was classified as antagonistic [23,24]. The numbers of synergistic, indifferent or antagonistic interactions were counted and their ratios were calculated.

Results were stratified into groups based on the MIC of azithromycin and ceftriaxone: ≤0.5 mg/L, or ≥1 mg/L for the azithromycin groups and ≤0.125 mg/L or ≥0.25 mg/L for the ceftriaxone groups. This stratification was designed to explore differences between sensitive and resistant isolates. The geometric means (GM) of MICs and FICIs were calculated. The non-parametric Mann-Whitney U test was implemented to examine the differences between MICs of three aforementioned antimicrobials in monotherapy and combination therapy.

## 3. Results

### 3.1. Antimicrobial Susceptibility Results of 380 N. gonorrhoeae Isolates

**Figure 1:**
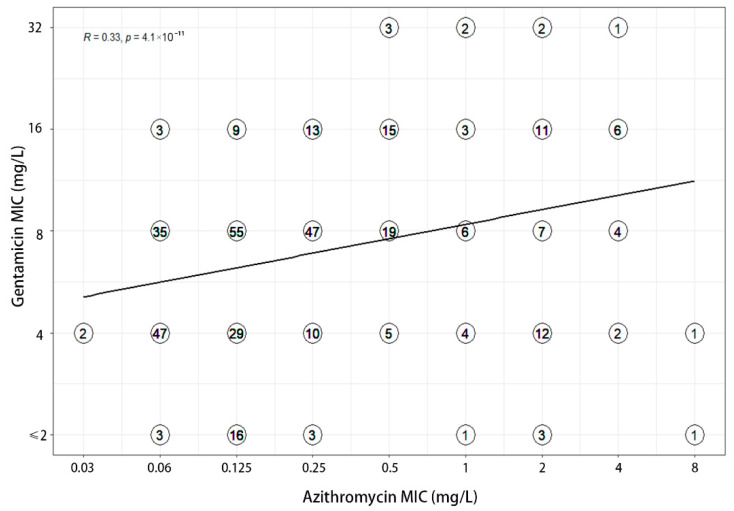
Correlation between MICs to gentamicin and azithromycin. Each symbol with a number signifies one or more isolates. Before computing the regression line, the MIC values were log transformed (log_2_).

**Figure 2:**
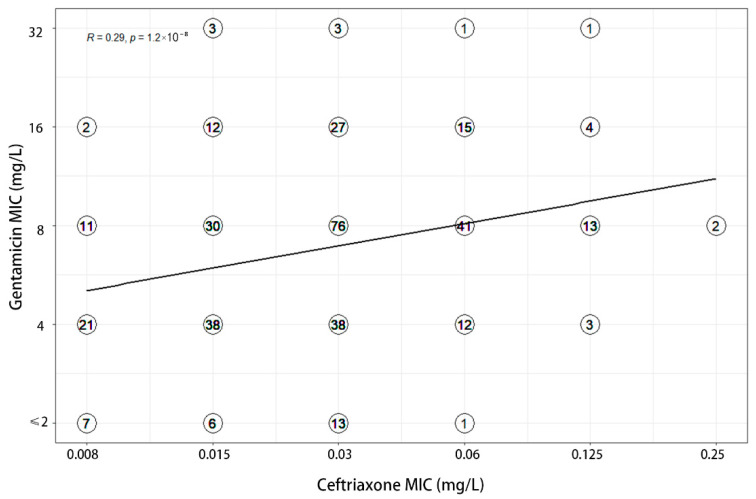
Correlation between MICs to gentamicin and ceftriaxone.

**Figure 3:**
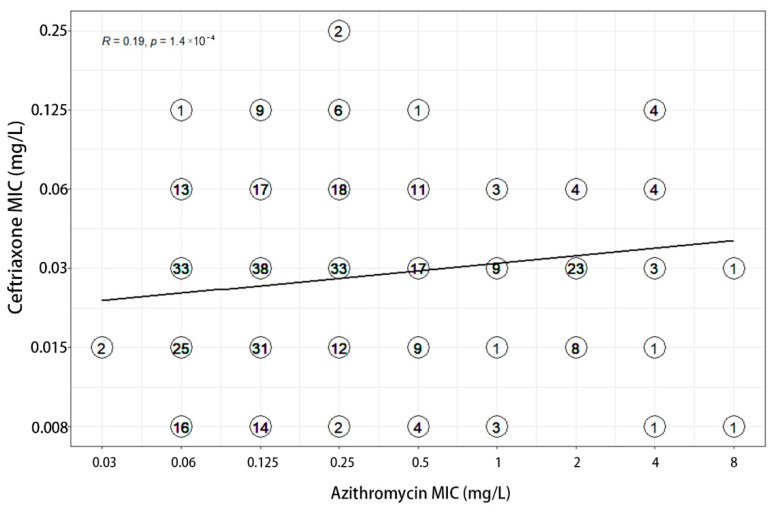
Correlation between MICs to azithromycin and ceftriaxone.

MIC values of three antimicrobials were recorded for each of the 380 clinical isolates (Table 1). MICs of gentamicin ranged from 1 to 32 mg/L, with MIC_50_ and MIC_90_ values of 8 and 16 mg/L respectively. A total of 139 (36.60%) and 233 (61.30%) isolates demonstrated full susceptibility and intermediate susceptibility to gentamicin, accordingly. Among the 66 azithromycin-resistant strains, 56.06% were intermediately susceptible to gentamicin and 36.36% were fully susceptible to gentamicin. The MIC values of gentamicin for both strains of ceftriaxone resistant bacteria were 4 mg/L. The correlation analysis showed that the MICs of ceftriaxone (R = 0.29) and azithromycin (R = 0.33) weakly correlated with that of gentamicin (Figure 1 and Figure 2). Furthermore, a weak correlation of r = 0.19 was also observed between azithromycin and ceftriaxone (Figure 3).

### 3.2. Synergy of Three Dual Antimicrobial Combinations

The results of three antibiotic combinations did not reveal any statistical disparities (Table 2). The mean FICI was 0.832 for the gentamicin and ceftriaxone combination, and 0.987 for the gentamicin and azithromycin combination. The synergistic effect of gentamicin with ceftriaxone was stronger than that of gentamicin with azithromycin, promoting 11.3% (43/380) of *N. gonorrhoeae* isolates. The combination of gentamicin and ceftriaxone exhibited antagonism only in 0.5% (2/380) strains and indifference in 88.2% (335/380) of strains. For the combination of gentamicin and azithromycin, 365 strains showed indifference, accounting for 96.1% of the total bacteria. For the treatment currently in use, the FICI value of ceftriaxone plus azithromycin is 0.874, which was also considered as no synergy or antagonism.

### 3.3. MICs of the Indicated Antibiotics as Monotherapy and in Combination

The GM MIC of gentamicin was 6.764 mg/L when tested alone; the GM MIC decreased to 2.327 mg/L (2.91-fold reduction, *p* < 0.05) when combined with ceftriaxone (Table 3). When tested alone, the MIC of ceftriaxone ranged from 0.008 to 0.25 mg/L; when combined with gentamicin, the GM MIC of ceftriaxone decreased from 0.027 to 0.014 mg/L (1.93-fold reduction, *p* < 0.05). The synergistic effect of gentamicin with ceftriaxone showed indifference regardless of whether the strain was resistant or sensitive to ceftriaxone.

The GM MIC of gentamicin combined with azithromycin decreased from 6.764 to 3.773 mg/L (1.79-fold reduction, *p* < 0.05). In monotherapy, the MIC for azithromycin ranged from 0.03 to 8 mg/L; yet combining with gentamicin led to a reduce of the azithromycin GM MIC from 0.226 to 0.072 mg/L (3.14-fold reduction, *p* < 0.05). Among azithromycin-resistant bacteria, the MIC values for gentamicin alone and in combination were 8.084 mg/L and 5.367 mg/L, respectively (1.51-fold reduction in combination compared to monotherapy). On the other hand, the GM MIC for azithromycin dropped from 2.021 to 0.314 mg/L (A 6.44-fold reduction, *p* < 0.05). The synergistic effect of gentamicin with ceftriaxone delineated no discrepancy regardless of whether the strain was azithromycin-resistant or sensitive.

The GM MIC of ceftriaxone decreased from 0.027 to 0.017 mg/L when combined with azithromycin (A 1.58-fold reduction, *p* < 0.05). The GM MIC for azithromycin decreased from 0.226 to 0.1 mg/L (A 2.26-fold reduction, *p* < 0.05). Overall, the combination did not produce any statistically significant difference.

## 4. Discussion

In this research, we studied the resistance profile of 380 clinical isolates of *N. gonorrhoeae* strains collected from three provinces in China with respect to gentamicin, azithromycin, and ceftriaxone. By the agar dilution method, we found that 36.60% of the isolates were fully susceptible to gentamicin, while 61.30% of the strains showed intermediate susceptibility. In two other studies from China on *N. gonorrhoeae* susceptibility to gentamicin, moderate susceptibility rates of 97.8% [25] and 91.6% [21] were also reported. The possible reason was that these two studies were both from eastern provinces of China, while the isolates of our study were from the eastern, northern and western regions of China. Through comparison with another study from seven provinces in 2016, a significant increment in gentamicin resistance was observed in this study, with the proportion of moderate susceptibility rates increasing from 14.0% to 61.3% and 2.1% of resistant isolates being identified [14]. This indicated an increasing trend of gentamicin resistance in China. Therefore, monitoring gentamicin resistance is still needed before implementing gentamicin for *N. gonorrhoeae* infections in China. It may be more appropriate to use gentamicin as an adjunct to combination therapy.

The antibiotic synergistic effects were studied using the agar dilution method. Several laboratory techniques can be applied to detect in vitro synergy between antibiotics, with the checkerboard and E test method being the most widely used techniques [26]. The checkerboard method includes microdilution, tube dilution and agar dilution. The agar dilution method is used to detect the MIC of combined antibiotics application by recording the growth of strains on plates with different concentrations of antibiotic combinations, while the E-test method is used to determine the MIC by placing E-test strips of the two antimicrobials on the agar plates at a 90° angle, with intersections at the points of their individual MICs [26,27,28]. Compared to the E-test method, the agar dilution method allows for the simultaneous detection of hundreds *N. gonorrhoeae* strains at the same antibiotic combination concentration. Therefore, agar dilution is suitable for studying a large number of isolates. According to a study of 65 dual antibacterial drug combinations, the E-test method and the agar dilution method did not result in a difference in the interpretation of synergy [22].

The synergistic effect of the combined antimicrobials is beneficial in obstructing the development of *N. gonorrhoeae* antibiotic resistance, and we evaluated the interaction of three antibiotics, of which azithromycin and ceftriaxone are the available treatments for gonorrhea and have different resistance mechanisms than gentamicin. We found no facilitative or antagonistic effects associated with these three combinations through evaluating the interactions. According to the data on antimicrobial combinations, three antibiotic combinations showed indifferent effects on most strains and demonstrated synergistic effects in a small number of strains. Several regional studies have reported FICI values ranging from 0.794 to 1.750 for gentamicin in combination with azithromycin [21,27,29,30], and 0.747 to 1.300 for gentamicin in combination with ceftriaxone [21,26,27,31]. These studies have shown that gentamicin-azithromycin and gentamicin-ceftriaxone combinations were indifferent in most clinical isolates.

In the subgroup study, it was observed that gentamicin was not synergistic or antagonistic with another antibiotic, either for the ceftriaxone or the azithromycin resistant or sensitive strains. Comparing the MIC values of antibiotic monotherapy and in combination, combined antibiotics application significantly reduced the previous MICs. Especially in azithromycin-resistant strains, the largest fold decrease (A 6.44-fold reduction) in MIC was observed for the azithromycin-gentamicin combination compared to azithromycin alone. For ceftriaxone, when combined with gentamicin, the GM MIC of ceftriaxone was reduced 1.93-fold. Thus, for these strains, combination therapy can contribute to the halting of the development of ceftriaxone and azithromycin resistance.

This study is clinically relevant and can offer a guide for the clinical application of gentamicin for the following reasons. Firstly, most of the *N. gonorrhoeae* isolates tested in this study were either fully or intermediately susceptible to gentamicin. As a nationwide study with multiple sample sizes, this study was more representative of the overall gentamicin resistance in China. A correlation analysis demonstrated weak correlations among the MICs of gentamicin, azithromycin and ceftriaxone, indicating the feasibility of using gentamicin in azithromycin or ceftriaxone resistant strains. Secondly, an interaction analysis illustrated the overall indifferent effects embodied by gentamicin in combination with azithromycin or ceftriaxone, providing a theoretical basis for multidrug combination therapy in resistant strains. Since dual combinations of three antibiotics demonstrated indifferent effects, this also provided ideas for future research related to the potential feasibility of a triple combination against clinically resistant strains.

However, there are some limitations in this study. First, only in-vitro experiments were conducted in this plan, hence differences in the penetration of various antibiotics in certain organs were out of the scope of this study. Moreover, according to pharmacokinetic studies, the half-life of azithromycin is 68 h, while that of gentamicin is 2 h [32,33]. Therefore, the combination dose of these two antibiotics is worthy of further investigation. Secondly, the amount of collected ceftriaxone-resistant bacteria used in this study is less than that of the other groups, which implies that more clinical isolates with a variety of resistant bacteria are needed to verify our findings.

## 5. Conclusions

In summary, most of the *N. gonorrhoeae* isolates tested in this study exhibited a certain degree of susceptibility to gentamicin. Antimicrobial combinations of gentamicin with ceftriaxone or azithromycin demonstrate indifferent effects overall; meanwhile the efficacy of individual antibiotics was enhanced in the presence of other antimicrobial agents. Gentamicin is generally effective in the treatment of gonorrhea and its combination with ceftriaxone or azithromycin can serve as an alternative therapeutic option.

## Figures and Tables

**Table 1:** *N. gonorrhoeae* isolates susceptibility to three antibiotics. (Gen, gentamicin).

Antimicrobial	Characterization	Number of Isolates for Which MIC to GEN Was					Total	Correlation to Gen
		≤2 mg/L	4 mg/L	8 mg/L	16 mg/L	32 mg/L		
Gentamicin	-	27	112	173	60	8	380	-
Azithromycin	Susceptible	22	93	156	40	3	314	R = 0.33
	Resistant	5	19	17	20	5	66	
Ceftriaxone	Susceptible	27	112	171	60	8	378	R = 0.29
	Resistant	0	0	2	0	0	2	

**Table 2:** Synergy testing results for three combinations (gentamicin plus ceftriaxone, gentamicin plus azithromycin, and ceftriaxone plus azithromycin) against 380 *N. gonorrhoeae* isolates.

Effect.	GEN and CRO	GEN and AZM	AZM and CRO
Synergistic [*n* (%)]	43 (11.3)	15 (3.9)	42 (11.1)
Indifferent [*n* (%)]	335 (88.2)	365 (96.1)	333 (87.6)
Antagonistic [*n* (%)]	2 (0.5)	0 (0)	5 (1.3)
FICI (geometric mean)	0.832 (0.370–4.5)	0.987 (0.280–3)	0.874 (0.280–6.167)
Classification overall	Indifferent	Indifferent	Indifferent

**Table 3:** MICs geometric mean and range of the indicated antibiotics as monotherapy and in combination.

	MIC Geometric Mean (Range) (mg/L)				FICI	Interpretation
	MIC_A_	MIC_A_ (with B)	MIC_B_	MIC_B_ (with A)		
GEN and CRO						
CRO MIC ≤ 0.125 mg/L (*n* = 378)	6.758 (1–32)	2.320 (1–16)	0.027 (0.004–0.125)	0.013 (0.004–0.125)	0.833 (0.37–4.5)	indifference
CRO MIC ≥ 0.25 mg/L (*n* = 2)	8	4	0.25	0.03	0.62	indifference
GEN and CRO all isolates (*n* = 380)	6.764 (1–32)	2.327 (1–16)	0.027 (0.008–0.25)	0.014 (0.004–0.125)	0.832 (0.370–4.5)	indifference
GEN and AZM						
AZM MIC ≤ 0.5 mg/L (*n* = 314)	6.515 (1–32)	3.504 (1–16)	0.143 (0.03–0.5)	0.052 (0.015–0.25)	1.004 (0.365–3)	indifference
AZM MIC ≥ 1 mg/L (*n* = 66)	8.084 (2–32)	5.367 (1–16)	2.021 (1–8)	0.314 (0.06–4)	0.919 (0.28–3)	indifference
GEN + AZM all isolates (*n* = 380)	6.764 (1–32)	3.773 (1–16)	0.226 (0.03–8)	0.072 (0.015–4)	0.987 (0.280–3)	indifference
AZM and CRO all isolates (*n* = 380)	0.226 (0.03–8)	0.1 (0.015–4)	0.027 (0.008–0.25)	0.017 (0.004–0.25)	0.874 (0.280–6.167)	indifference

## Data Availability

The data presented in this study are available in the article.
